# *Helicobacter*, Hygiene, Atopy, and Asthma

**DOI:** 10.3389/fmicb.2017.01034

**Published:** 2017-06-08

**Authors:** Muhammad Miftahussurur, Iswan A. Nusi, David Y. Graham, Yoshio Yamaoka

**Affiliations:** ^1^Gastroenterology and Hepatology Section, Department of Medicine, Baylor College of MedicineHouston, TX, United States; ^2^Department of Environmental and Preventive Medicine, Oita University Faculty of MedicineYufu, Japan; ^3^Gastroentero-Hepatology Division, Department of Internal Medicine, Faculty of Medicine-Institute of Tropical Disease, Universitas AirlanggaSurabaya, Indonesia

**Keywords:** *Helicobacter pylori*, hygiene hypothesis, asthma, atopy, allergy

## Abstract

The hygiene hypothesis links environmental and microbial exposures in early life to the prevalence of atopy, allergy, and asthma. *Helicobacter pylori* infection is typically acquired in childhood and acquisition of the infection is associated with poor household hygiene. Some population surveys have shown an inverse association between *H. pylori* infection and atopy, allergy, and asthma leading to the suggestion that *H. pylori* infection may be protective against disease; others consider it simply a biomarker for poor household hygiene. We review the relevant surveys, cohort studies, meta-analyses, and studies testing the protective hypothesis. Overall, the results of surveys and cohort studies are inconsistent, whereas meta-analyses show a significant but weak inverse correlation. In contrast, studies directly testing the protection hypothesis in relation to asthma in populations with poor hygiene and low *H. pylori* prevalence failed to confirm a protective effect. *H. pylori* is a major cause of human disease including chronic gastritis, peptic ulcer, and gastric malignancies. *H. pylori* infections most likely serve as a biomarker for poor hygienic conditions in childhood. We conclude that while synergistic interactions between environmental factors in childhood are important determinants of the pathogenesis of atopy, allergy, and asthma; *H. pylori* is inversely related to good hygiene and thus it's presence serves as a biomarker rather than for a specific prevention role for *H. pylori* or *H. pylori* antigens.

## Introduction

Until recently, the prevalence of asthma (Gershon et al., 2010; de Marco et al., 2012), rhinitis (Hansen et al., 2013), and atopic dermatitis (Duggan et al., 2012) has been increasing in many developed countries. This increase coincided with improved hygiene and socioeconomic conditions and with a decrease in the incidence of many infectious diseases (Bach, 2002) as well as with an increase in the consumption of fossil fuels (Shafiee and Topal, 2009). Although earlier researchers had proposed theories linking atopic disease and hygiene (Blackley, 1873; Leibowitz et al., 1966), in 1989 Strachan proposed a formal theory (i.e., the “hygiene hypothesis”) based on studies of the relationship between hay fever and microbial infections in early childhood and adolescence in the United Kingdom (Strachan, 1989). In essence, the hypothesis holds that improved hygiene in early life reduces microbial exposures which were important in priming the immune response and were protective against atopic disorders. This hypothesis was subsequently expanded to cover asthma and autoimmune diseases (Okada et al., 2010).

*Helicobacter pylori* is a gram-negative spiral bacterium etiologically associated with both gastric and extragastric diseases including gastric cancer (Graham, 2015). Although the incidence and prevalence of *H. pylori* has markedly decreased in many developed countries, overall at least 50% of adults worldwide are infected (Leja et al., 2016). The infection is typically acquired in childhood. Because the increase in childhood atopic diseases appeared to correspond to a fall in *H. pylori* acquisition, it was suggested that the two conditions might be related. Several mechanisms were proposed to link the hygiene hypothesis with *H. pylori* infections (Cremonini and Gasbarrini, 2003; Shiotani et al., 2008). *H. pylori* infection is thought to influence the process of inducing naïve T cells in the two main functional groups: T helper 1 (Th1) and helper 2 (Th2) subsets. For example, T cells in the gastric mucosa of *H. pylori*-infected patients produce relatively more interferon-γ and relatively less IL-4 than is found in the gastric mucosa of uninfected individuals suggesting that that *H. pylori* may lead to a Th1-polarized immune response (Bamford et al., 1998; Sommer et al., 1998). Accordingly, *H. pylori* infection may also reduce the risk of asthma and allergy due to suppression of the Th2 response (Fox et al., 2000).

T-regulatory (Treg) cells are also increased in *H. pylori-*infected human gastric mucosa (Lundgren et al., 2005). Experiments in mice showed that the persistence of *H. pylori* was associated with reprograming of dendritic cell resulting in impairment of T-cells effector function, induction of mucosal T-reg cells and skewing of the immune response toward tolerance (Oertli et al., 2012). Two *H. pylori* antigens (γ-glutamyl transpeptidase and VacA) induced Treg cells in the mouse gastric mucosa resulting in development of tolerance and a reduction in allergic responses (Oertli et al., 2013). Of interest, induction of Treg in the gastric mucosa is also an important step in the establishment and maintenance of *H. pylori-*induced gastric adenocarcinoma (Kandulski et al., 2008, 2010). Although animal studies clearly showed a relation between experimental *H. pylori* infection and protection from ovalbumin-induced asthma (Codolo et al., 2008; Arnold et al., 2011), the neonatal mouse model provides similar results with many different antigens other than those associated with *H. pylori* (Fujimura et al., 2014). Thus, while the results with neonatal mice are reproducible, the marked differences between the immune system of neonatal mice and humans suggest the need for great caution when trying to apply the lessons learned in neonatal mice to human disease (Renz et al., 2012).

Clinical studies in Taiwan have shown that mass eradication of *H. pylori* can remarkedly reduce the incidence of peptic ulcer, gastric cancer and gastric atrophy (Lee Y. C. et al., 2013). *H. pylori* eradication was also shown to reduce the risk of metachronous cancer after endoscopic treatment of primary gastric cancer (Yoon et al., 2014). While there is current interest in worldwide *H. pylori* eradication to reduce or eliminate gastric cancer (Graham and Uemura, 2006; Graham, 2015; Lee et al., 2016), it has been suggested that an eradication program might have untoward effects if *H. pylori* has a protective role against asthma and atopic diseases (Noverr et al., 2005).

Although most *H. pylori* infections are asymptomatic about 20% eventuate in a potentially life threatening clinical disease. The fact that progressive gastric damage is often silent has suggested to some that the bacterium may be harmless, commensal, or even beneficial (Carroll et al., 2004; Mishra, 2013). We collected data from PubMed using keyword combination *H. pylori* (*pylori* or *Helicobacter*) with atopy (atopy or atopic disease or atopic dermatitis), allergy (allergy, allergic disease, allergic rhinitis) and asthma for articles published through March 2016. We excluded abstracts alone or unpublished articles. Here, we summarize the controversies surrounding the role of gastric *H. pylori* colonization as protective against atopy, allergy, and asthma or whether the presence of *H. pylori* is primarily that of a biomarker for poor hygiene.

## Association between *H. pylori* infection and atopy, allergy, and asthma

### Positive studies

Surveys in Europe, America, and Asia have reported an inverse relationship between *H. pylori* infection and atopy, allergy, and asthma (Kosunen et al., 1997; Chen and Blaser, 2007; Shiotani et al., 2008). Two cross-sectional studies have been based on National Health and Nutrition Examination Surveys in the United States (Chen and Blaser, 2007, 2008) (Table 1). The initial study involved 7,412 adults (NHANES III) and reported that *H. pylori* infection was significantly and inversely associated with dermatitis, rash, and eczema in the last year leading up to the survey (OR 0.73; 95% CI 0.56–0.96). Similar trends were observed for asthma (OR 0.89; 95% CI 0.68–1.16) and wheezing (OR 0.73, 95% CI 0.57–0.94) (Chen and Blaser, 2008). Moreover, *H. pylori* was inversely associated with past (OR 0.69, 95% CI 0.45–1.06) or current bouts of asthma (OR 0.41, 95% CI 0.24–0.69), and with recent episodes of wheezing, allergic rhinitis, dermatitis, eczema, and rash (Chen and Blaser, 2008). The other survey of 7,663 adults (NHANES 1999–2000) with asthma, allergic rhinitis, and atopic disorder by the same group (Chen and Blaser, 2007) also revealed that antibody to the *H. pylori* cytotoxin-associated gene product (CagA), a marker of more inflammatory type of *H. pylori* infection, was inversely correlated with asthma (OR 0.79; 95% CI 0.63–0.99) and allergic rhinitis (OR 0.77; 95% CI 0.62–0.94), especially among those who developed these conditions in childhood (Table 1; Study No. 10). Moreover, individuals (median age <43 years) who tested positive for for CagA and *H. pylori* antibodies were less likely to have developed allergies in the last year leading up to the survey and were less likely to be sensitized to pollens and molds, compared to antibody negative individuals (Chen and Blaser, 2007). Based on these two studies, *H. pylori* infection was proposed to possibly be protective against asthma and allergy. A subsequent case-control study of 318 adults with asthma and 208 controls in New York (Reibman et al., 2008) also reported the presence of CagA antibody to be significantly and inversely correlated with asthma (OR 0.57, 95% CI = 0.36–0.89) after adjustment for age, race, and income. In addition, a survey in an adult population in the UK based on urea breath testing for active *H. pylori* infection found a 30% reduction in the risk of atopy (McCune et al., 2003). Finally, levels of IgE antibodies were found to be higher in adult Finnish individuals who also had *H. pylori* antibodies in 1994 but not in 1973 (Kosunen et al., 2011). Similar results were reported in Danish (Linneberg et al., 2003), Israeli (Zevit et al., 2012), Scandinavian (Janson et al., 2007), and Japanese (Shiotani et al., 2008) populations.

**Table 1:** Association between *H. pylori* and atopy, allergy, and asthma in cross-sectional studies.

**No**.	**Author**	**Year**	**Study period**	**Location**	**Mean age (range)**	***H. pylori* diagnosis**	**Indicator**	***H. pylori* positive/Indicator positive (%)**	***H. pylori* positive/Indicator negative (%)**	***P*-value**
1	Kosunen	2002	1973	Finland	(15–54)	IgG and IgA *H. pylori*	IgE	16/147 (10.9)	20/179 (11.2)#	0.9340
			1994					3/59 (5.1)¶	54/260 (20.8)#	0.0095
2	Linneberg	2003	1990–1991	Denmark	(15–69)	IgG *H. pylori*	IgE	75/273 (27.5)¶	323/824 (39.2)#	<0.001¶¶
							Allergic rhinitis by questionnaire	48/271 (17.7)¶	236/822 (28.7)#	<0.001¶¶
3	Cullinan	2003	1198–1999	England	28	IgG *H. pylori*	Skin prick test	53/151 (35.0)¶	278/745 (37.0)#	0.52
4	McCune	2003	N/A	England	(20–59)	Urea breath test	Atopy by questionnaire	85/1079 (7.9)¶	235/2165 (10.9)#	0.007
5	Jarvis	2004	1992–1993	England	(20–44)	IgG *H. pylori*	Wheeze by questionnaire	60/208 (28.9)¶	167/613 (27.2)#	0.64
							Waking with cough by questionnaire	62/208 (30.0)¶	190/613 (31.0)#	0.21
							Hay fever by questionnaire	60/208 (28.9)¶	181/613 (29.6)#	0.95
							IgE	83/208 (39.9)¶	230/613 (37.6)#	0.43
6	Radon	2004	2002	Germany	(18–44)	IgG *H. pylori*	IgE	18/91 (19.8)	50/230 (21.7)	0.814¶¶
7	Pessi	2004	N/A	Finland	>30	IgG *H. pylori*	Asthma by physician	115/245 (46.9)	205/405 (50.6)	0.370
8	von Hertzen	2005	1997–1998	Finland	25–54	IgG *H. pylori*	Skin prick test	62/268 (23.1)	141/507 (27.8)	0.526
				Russia	25–54	IgG *H. pylori*	Skin prick test	78/90 (86.5)	280/297 (94.3)	0.011
9	Jun	2006	2005–2005	China	50.5	IgG *H. pylori*	Chronic bronchitis	40/46 (86.9)	29/48 (60.4)	<0.01
10	Chen	2007	1998–1994	United States	43.0	IgG *H. pylori*	Self-reported asthma (current)	169/3,720 (4.5)¶	196/3,943 (5.0)#	0.409¶¶
							Self-reported asthma (lifetime)	229/3,720 (6.2)¶	296/3,943 (7.5)#	0.022¶¶
							Self-reported allergic rhinitis (current)	236/3,720 (6.3)¶	380/3,943 (9.6)#	<0.001¶¶
							Self-reported allergic rhinitis (lifetime)	278/3,720 (7.5)¶	439/3,943 (11.1)#	<0.001¶¶
							Self-reported allergic symptoms	1,935/3,720 (52.0)¶	2,398/3,943 (60.8)#	<0.001¶¶
11	Seiskari	2007	1994, 1997–1999	Finland, Russia	11.4 (7–15)	IgG *H. pylori*	IgE	9/194 (4.6)¶	8/72 (11.1)#	0.055
12	Chen	2008	1999–2000	United States	(14–49)	IgG *H. pylori*	Asthma by questionnaire (ever)	267/2,625 (10.2)¶	679/4,787 (14.2)#	0.05
							Dermatitis, eczema, rash by questionnaire	234/2,625 (8.9)¶	514/4,787 (10.7)#	0.014¶¶
							Wheeze by questionnaire	275/2,625 (10.5)	653/4,787 (13.6)#	<0.001¶¶
					(3–19)	IgG *H. pylori*	Allergic rhinitis by questionnaire	62/750 (8.3)¶	275/2,577 (10.7)#	0.02
							Asthma by questionnaire (current)	66/750 (8.8)¶	253/2,577 (9.8)#	0.03
							Asthma by questionnaire (ever)	98/750 (13.1)¶	409/2,577 (15.9)#	0.15
							Allergic rhinitis and asthma (ever)	14/750 (1.9)¶	81/2,577 (3.1)#	0.99
13	Baccioglu	2008	N/A	Turkey	38.0 (17–74)	Histopathology	Skin prick test	20/74 (27.0)¶	4/16 (25.0)#	0.86
							Asthma by physician	8/74 (10.8)¶	5/16 (31.3)#	0.03
							Rhinitis	45/74 (60.8)¶	11/16 (68.8)#	0.77
							Urticaria	20/74 (27.0)¶	6/16 (37.5)#	0.54
							Food allergy	9/74 (12.2)¶	1/16 (6.3)#	0.49
14	Fullerton	2009	1991	England	(18–71)	IgG *H. pylori*	Skin prick test	162/643 (25.2)¶	552/1,732 (31.9)#	0.002¶¶
							Hay fever	143/643 (22.2)¶	455/1,732 (26.3)#	0.05¶¶
							Chronic bronchitis	107/643 (16.6)¶	208/1,732 (12.0)#	0.004¶¶
							Asthma by physician	62/643 (9.6)¶	151/1,732 (8.7)#	0.536¶¶
15	Zevit	2012	2007–2008	Israel	(5–18)	Urea breath test	Asthma by physician	233/3,175 (7.3)¶	345/3,784 (9.1)#	0.007
16	Karimi	2013	2010–2011	Iran	(6–12)	Urea breath test	Asthma by physician	18/98 (18.4)	23/98 (23.4)	0.380
17	Lee	2014	2010–2013	Korean	55.4	IgG *H. pylori*	Asthma by questionnaire and IgE	225/320 (70.3)	1,159/1,794 (64.6)	0.667¶¶
18	Hollander	2016	2002–2006	Netherland	6	IgG *H. pylori*	Wheezing by questionnaire	18/269 (6.7)	231/2,866 (8.1)	0.24
							Asthma by questionnaire	23/198 (11.6)	219/2,864 (7.6)	0.045
							Eczema by questionnaire	59/610 (9.7)	189/2,472 (7.6)	0.25
19	Lim	2016	2011	South Korea	≥18	IgG *H. pylori*	Asthma by physician, questionnaire	229/9,492 (2.4)¶	130/5,540 (2.3)#	0.333¶¶

¶ Atopy, allergy, or asthma positive/H. pylori positive;

# *Atopy, allergy, or asthma positive/H. pylori negative*.

¶¶ *When authors did not provide P-values, we calculated them using SigmaStat version 3.5 (Systat Software, Inc., Richmond, CA)*.

### Negative studies

Contradictory results include a study in which *H. pylori* failed to protect Finnish siblings equally from atopic sensitivity (53/151, 35% vs. 278/745, 37%, *P* = 0.52) (Cullinan et al., 2003). Higher IgE antibodies were not associated with *H. pylori* antibodies in Germans (Radon et al., 2004). A survey of 240 atopic patients and 240 controls from a larger cohort of 1,659 Italians (Matricardi et al., 2000) (Table 2) did not find significant associations, although the adjusted OR for atopy decreased along a gradient of exposure to *H. pylori* (35/240, 15% vs. 44/240, 18%, *P* = 0.325). A skin prick test for allergy also failed to discriminate between Turkish volunteers infected with or uninfected with *H. pylori* (Baccioglu et al., 2008), and between those with or without asthma (Annagur et al., 2007). *H. pylori* antibody prevalence was also higher in Chinese (Tsang et al., 2000) and Japanese (Jun et al., 2005) patients with asthma, but not significantly. In 1,211 subjects randomly selected from 15,000 British adults (Jarvis et al., 2004), the prevalence of cough and hay fever was similar in both infected and uninfected individuals (62/208, 30% vs. 190/613, 31%, *P* = 0.21 and 60/208, 28.9% vs. 181/613, 29.6%, *P* = 0.95, respectively). Wheezing (*P* = 0.64) and allergen-specific serum IgE (*P* = 0.43) were also higher in *H. pylori-*seropositive adults, but not statistically. Based on this study Jarvis et al. (2004) concluded that the evidence did not link *H. pylori* to cough, hay fever, and wheezing. A survey in China also showed that *H. pylori* and CagA IgG antibodies were more prevalent in patients with chronic bronchitis than in controls (Jun et al., 2006). Finally, a 30-year nested case-control study of 113 participants with adult-onset wheezing and 267 controls in Scotland (Bodner et al., 2000) (Table 2) found that atopic disorders were not significantly associated with *H. pylori* infection. The frequency of wheezing symptoms was comparable between groups, and was weakly but positively correlated with *H. pylori* antibodies. Furthermore, chronic cough and phlegm were more likely to be reported in *H. pylori* seropositive volunteers (6/19, 23.1%) than in seronegative participants (18/150, 12%, *P* ≤ 0.05). The latest studies also showed the contradictory that subjects with past *H. pylori* infection had significantly a highest prevalence of allergy based on IgE status (Lee et al., 2015) and a higher prevalence recent astma in *H. pylori* positive than *H. pylori* negative children (den Hollander et al., 2016).

**Table 2:** Association between *H. pylori* and atopy, allergy, and asthma in case-control studies.

**No**.	**Author**	**Year**	**Study period**	**Location**	**Mean age (range)**	***H. pylori* diagnosis**	**Indicator**	***H. pylori* positive/Indicator positive (%)**	***H. pylori* positive/Indicator negative (%)**	***P*-value**
1	Matricardi	2000	1990–1991	Italy	(17–24)	IgG *H. pylori*	IgE	35/240 (15.0)	44/240 (18.0)	0.325
2	Bodner	2000	1995	Scotland	(39–45)	IgG *H. pylori*	IgE	77/150 (51.3)	65/125 (52.0)	0.991¶¶
							Wheeze	49/85 (57.6)	93/190 (48.9)	0.229¶¶
							Chronic cough and phlegm	6/19 (23.1)	18/150 (12.0)	0.05
3	Tsang	2000	1997–1998	Hong Kong	42.6	IgG *H. pylori*	Asthma by physician	44/90 (48.9)	37/97 (38.1)	0.30
4	Jun	2005	2004–2005	Japan	51.2	IgG *H. pylori*	Asthma by physician	10/46 (21.7)	9/48 (18.8)	0.917¶¶
5	Jaber	2006	2001–2003	Saudi Arabia	1 to ≥ 10	IgG *H. pylori*	Asthma by physician	45/220 (20.4)	128/543 (23.6)	0.36
6	Annagur	2007	2003–2005	Turkey	(5–15)	IgG *H. pylori*	Asthma by physician	20/79 (25.3)	6/36 (16.7)	0.227
7	Janson	2007	1990–1994	Estonia, Iceland, Sweden	(20–44)	IgG *H. pylori*	IgE	81/327 (24.8)	337/922 (36.6)	<0.001
8	Shiotani	2008	2005–2006	Japan	19.5	IgG *H. pylori*	Allergy by questionnaire	42/369 (11.4)	72/408 (17.6)	0.015
9	Reibman	2008	N/A	United States	(18–65)	IgG *H. pylori*	Asthma by questionnaire	147/318 (46.2)	100/208 (48.1)	0.744¶¶

¶¶ *When authors did not provide P-values, we calculated them using SigmaStat version 3.5 (Systat Software, Inc., Richmond, CA)*.

### Cohort studies

Three cohort studies have been reported (Cam et al., 2009; Amberbir et al., 2011; Holster et al., 2012) (Table 3). In one study, 74 children in Turkey were followed for 6 years and (Cam et al., 2009) found that atopy was less prevalent, but not significantly, in *H. pylori*-infected children (39.1 vs. 48.1%, *P* = 0.215). Similarly, a 3-year study on a cohort of Ethiopian children revealed that of 832 children infected with *H. pylori*, 235 (28.2%) did not experience hay fever, a prevalence that was not significantly lower than in uninfected children (18/44, 40.9%, *P* = 0.09). Similar trends were observed for wheezing. Nevertheless, borderline significant protection against eczema was noted (OR 0.49, 95% CI 0.24–1.01, *P* = 0.05). *H. pylori* was inconsistently associated with asthma and allergy in the third study and was not considered either beneficial or protective (Holster et al., 2012).

**Table 3:** Association between *H. pylori* and atopic, allergy, and asthma in cohort studies.

**No**.	**Author**	**Year**	**Study period**	**Location**	**Mean age (range)**	***H. pylori* diagnosis**	**Indicator**	***H. pylori* positive/Indicator positive (%)**	***H. pylori* positive/Indicator negative (%)**	***P*-value**
1	Cam	2009	1999–2015	Turkey	14.8	Urea breath test	Skin prick test	15/47 (31.9)¶	13/27 (48.1)#	0.215
							Asthma by questionnaire	4/47 (8.5)¶	1/27 (3.7)#	0.646
							Allergic rhinitis	3/47 (6.4)¶	1/27 (3.7)#	1.00
							Atopic eczema	3/47 (6.4)¶	1/27 (3.7)#	1.00
2	Amberbir	2011	2005–2009	Ethiopia	3.0	Stool antigen	Self-reported wheeze	24/80 (30.0)	229/796 (28.8)	0.41
							Self-reported eczema	11/55 (20.0)	242/821 (29.5)	0.05
							Self-reported hay fever	18/44 (40.9)	235/832 (28.2)	0.09
							Self-reported *D. pteronyssinus*	6/48 (12.5)	247/816 (30.3)	0.07
							Self-reported cockroach	6/36 (16.7)	247/828 (29.8)	0.29
3	Holster	2012	1996–2004	Netherlands	(7–9)	IgG *H. pylori*	Wheeze by questionnaire	12/204 (5.9)	37/341 (10.9)	0.05
							Allergic rhinitis by questionnaire	25/294 (8.5)	24/251 (9.6)	0.779¶¶
							Atopic dermatitis by questionnaire	21/241 (8.7)	28/304 (9.2)	0.960¶¶
							Physician-diagnosed asthma by questionnaire	7/98 (7.1)	42/447 (9.4)	0.609¶¶

¶ Atopy, allergy, or asthma positive/H. pylori positive;

# *Atopy, allergy, or asthma positive/H. pylori negative*.

¶¶ *When authors did not provide P-values, we calculated them using SigmaStat version 3.5 (Systat Software, Inc., Richmond, CA)*.

*H. pylori* infection is significantly associated with drinking water, toilet type, location of residence, number of residents in a household, and birth order (Malaty et al., 1996; Nurgalieva et al., 2002; Ueda et al., 2003). These associations are well-demonstrated in a cross sectional study with two geographically contiguous cohorts of 266 Finnish and 266 Russian Karelian children living in markedly different cultures, economic systems, and standards of living (e.g., gross domestic product per capita US$ 32,790 vs. US$ 3,410) (Seiskari et al., 2007). These children were also surveyed for other microbes associated with poor hygiene, including hepatitis A virus, *Toxoplasma gondii*, and enterovirus. Importantly, antibodies against all microbes surveyed were higher in Russian Karelian children than in Finnish children. Conversely, allergen-specific IgE was higher in the latter than in the former, but allergy and atopy were not associated with *H. pylori* infection in Finnish children.

### Meta-analyses

Because cohort studies were inconclusive as to whether *H. pylori* might either be protective or a surrogate for the hygiene hypothesis (Table 3), meta-analyses were performed to increase the statistical power by critically appraising and synthesizing data from multiple studies (Goodman et al., 2015). Meta-analyses are available regarding a possible link between *H. pylori* infection and atopy (Lionetti et al., 2014; Taye et al., 2015) or asthma (Wang et al., 2012, 2013; Zhou et al., 2013). Zhou et al. pooled data from 14 studies (28,283 patients) and found that *H. pylori* infection was significantly less frequent among volunteers with asthma than among controls (OR 0.84; 95% CI 0.73–0.96, *P* = 0.013) (Zhou et al., 2013). However, the differences were only significant in North America but not in the Asia and Europe or in a subanalysis related to positivity of CagA. Another meta-analysis noted a significant inverse association between *H. pylori* and asthma in both cross-sectional studies of 30,239 subjects (OR 0.84; 95% CI 0.74–0.96) and cohort studies of 1,235 subjects (OR 0.82; 95% CI 0.53–1.27). In case-control studies (2,544 subjects) there was a weak inverse association (OR 0.94; 95% CI 0.79–1.12) (Wang et al., 2013). An 18% reduction of the risk (OR 0.82; 95% CI 0.73–0.91, *P* = 0.01) for atopy was also observed among *H. pylori*-infected participants in a meta-analysis of 21,348 participants that combined various study designs (i.e., mixed analysis of cross sectional and cohort studies) (Taye et al., 2015). Of note, a meta-analysis of five case-control (1,555 participants) studies did not confirm an inverse (OR 1.01, 95% CI 0.82–1.24) between *H. pylori* and asthma (Wang et al., 2012). A meta-analysis that included asthma within the atopy/allergy cases found that allergy and atopy had a significant inverse association with *H. pylori* in 11 case-control studies of 4,607 participants (OR 0.80, 95% CI 0.62–0.97) whereas only allergy was inversely correlated in the cross-sectional studies of 14,198 participants (OR 0.74, 95% CI 0.65–1.16) (Lionetti et al., 2014).

These meta-analyses are not without problems as they generally aggregrated multiple heterogeneous conditions into a single group (i.e., different criteria for asthma diagnosis such as physicians vs. symptoms) and, except for few studies, used serological detection of *H. pylori*. Serology does allow one to differentiate between recent and past infections. In addition, the meta-analyses were designed to examine the association between a single agent, *H. pylori* with asthma, allergy and atopy without considering whether it was specifically involved or was acting as a weak surrogate for the hygiene hypothesis.

## Prospective studies testing the protection hypothesis

Overall, the prevalence of asthma appears to have increased earlier due in part to improvement in diagnostic and awareness. This increase now appears to have peaked and globally the prevalence of asthma has been reported to be decreasing or to have plateued (i.e., in Greece, Turkey, Scotlandia, and England) (Sears, 2014). For example, *H. pylori* infection has steadily decreased in England (Vyse et al., 2002) and if *H. pylori* were protective, one would expect the incidence of asthma to increase. However, asthma has decreased in all groups. particularly in children under 5 years (Simpson and Sheikh, 2010). Even more striking is the fact that those children least likely to have *H. pylori* (i.e., the higher socioeconomic class) experienced a greater fall in asthma incidence than seen in the lower socioeconomic classes (in which the prevalence also fell) (Graham, 2015).

The hypothesis that *H. pylori* is protective is based on improved hygiene which reduced *H. pylori* infections leading to increased risk. This hypothesis can be prospectively evaluated by examination of the prevalence of atopic/allergic disease in populations with low *H. pylori* prevalence but low incomes and hygienic standards. Such populations allow one to separate low prevalence of *H. pylori* infection from reduced hygiene and directly attempt to falsify the hypothesis that *H. pylori* provides the protective antigens responsible for protection against disease, in this case asthma.

**Figure 1:**
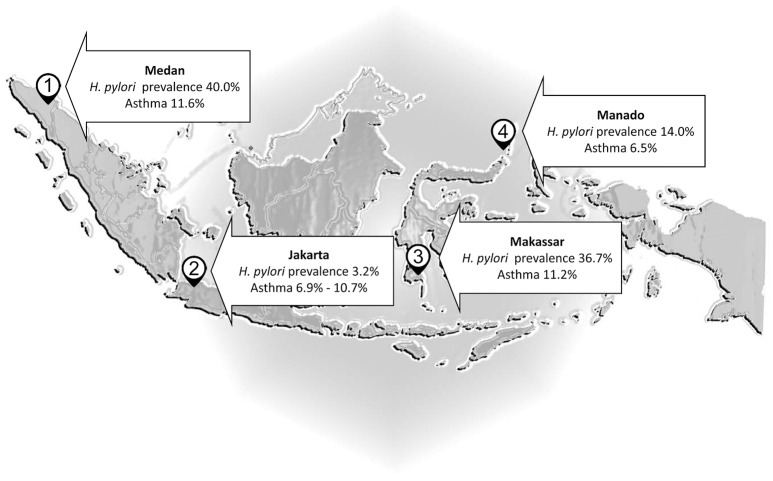
*Helicobacter pylori* and asthma in Indonesia. The prevalence of asthma is almost similar in Medan, Makassar, and Jakarta although the prevalence *H. pylori* infection is higher in Medan and Makassar than Jakarta.

There are a number of countries where *H. pylori* infections are rare despite poor living conditions (e.g., Malaysia, Indonesia, and Zanzibar) (Farag et al., 2007; Lee Y. Y. et al., 2013; Syam et al., 2015). For example, *H. pylori* infection is infrequent in Malaysia (Uyub et al., 1994; Sasidharan and Uyub, 2009) and thus one would expect a high rate of any disease against which *H. pylori* offered protection. However, wheezing due to asthma occurs in only 4.3% of Malaysian children 6–7 years old and 5.7% of those 13–14 years old. Allergic rhinitis in primary school is also rare (5%) (Quah et al., 2005) and asthma in the general population was noted to be relatively low in comparison to 56 other countries (Raj et al., 2009). In Indonesia (Figure 1), there was no an inverse correlation between the frequency of asthma and *H. pylori* infection: asthma affected 6.9% in Jakarta (*H. pylori* prevalence 3.2%) vs. 6.5% of the population in Manado (*H. pylori* prevalence 14%) (Sundaru, 2005; Syam et al., 2015). The prevalence of asthma is similar in Medan (11.6%), Makassar (11.2%), and Jakarta (10.7%) although the prevalence *H. pylori* infection is about 12 and 11 times higher in Medan and Makassar than Jakarta, and is about 28 and 23 times higher in Batak and Buginese than Javanese as the predominant ethnics in Medan, Makassar, and Jakarta, respectively (Sundaru, 2005; Syam et al., 2015). Thus, the original projections regarding asthma in countries with low *H. pylori* prevalence and relatively poor hygiene failed.

## The hygiene hypothesis and *H. pylori* infection

The hygiene hypothesis suggests that environmental and microbial exposure can shape the developing immune system and confer protection or risk against subsequent immune-mediated disease (von Mutius, 2007). Accordingly, commensal saprophyte microflora or probiotic bacteria with various microbial antigens may have a role in reducing the individual risk of allergy (e.g., living or a farm or having a puppy) (Renz et al., 2012; Campbell et al., 2016). In both Estonia and Sweden, where the prevalence of allergy is widely different (Bjorksten et al., 1999), patients with allergy harbored less lactobacilli and bifidobacterium than controls, regardless of exposure to antibiotics. Of note, Estonian children with allergy harbored a higher number of aerobic microorganisms, especially coliforms, than *Staphylococcus aureus*, which was dominant in allergic Swedish children. However, another study reported that lactic acid bacteria and bifidobacterium were comparable between wheezing patients sensitized or not sensitized in childhood (Murray et al., 2005).

Sheikh and Strachan (Sheikh and Strachan, 2004) clarified that the hygiene hypothesis is “framed in conceptual rather than specific terms.” Therefore, the protective effects of poor hygiene is likely due to synergism of multiple factors in the host and environment, rather than to a single agent (Vercelli, 2006). For example, adolescents who grow up on farms are less prone to asthma and allergic rhinitis than those who do not, possibly related to consumption of farm milk with higher concentrations of gram-negative bacteria and lipopolysaccharides (Riedler et al., 2001; Campbell et al., 2016). A recent meta-analysis across 14 countries (10,201 participants) also confirmed the role of the farm environment as a protective allergic risk and marker of microbial diversity for the inner city population (Campbell et al., 2016). Farms abound in microbial products, molds, and fungi and daily contact with these drive the maturation of immunity and reduce the risk of atopy and asthma (von Mutius, 2007). The prevention approach which was proposed as primary and secondary prevention of allergic diseases also emphasized not only a single agent, but multifactorial (i.e., hypoallergenic formula and reducing several allergens exposure) (Halken, 2004). Finally, it is also possible that protective events occur prior to childhood. For example, repeated pregnancy or intrauterine environment are associated with atopy (von Mutius, 2007).

Overall, allergy and asthma are considered multifactorial diseases triggered by interactions between the environment and host genes (McLeish and Turner, 2007). In general, allergic disorders are more prevalent in the northern than in the southern hemisphere, particularly in developed countries, in which rapid environmental changes in the last decades has led to increased outdoor and indoor pollution, climate change, and improved hygiene (Lionetti et al., 2014). Nonetheless, the hygiene hypothesis is not universally applicable, such as in the community with the lowest per capita income in New York. In this community, Afro-American and Hispanic-American children live in households with poor standards of living and hygiene, but exposure to various allergens and viruses paradoxically trigger asthma hospitalizations, with mortality rates more than five times higher than the national average (Webber et al., 2002; Garn and Renz, 2007).

## Is *H. pylori* infection beneficial?

The ancestor of *H. pylori* is believed to have established an ecological niche in the human stomach at least 100,000 years ago and then co-evolved with the host (Covacci et al., 1999). Nevertheless, its long history in the human stomach provides no information regarding whether *H. pylori* infection might be beneficial (Carroll et al., 2004; Mishra, 2013). For instance, hepatitis B virus is not considered commensal nor potentially beneficial, even though it accompanied humans from Africa 100,000–150,000 years ago (Robertson and Margolis, 2002). Hepatitis B infects one third of the global population, of whom more than 350 million are chronically infected. Like *H. pylori* the majority remain asymptomatic while the disease progresses with a small proportion presenting with complicated cirrhosis or hepatocellular carcinoma (El-Serag, 2012). Similarly, *Mycobacterium tuberculosis* continues to be a target of eradication even today, particularly in developing countries, even though it accompanied humans out of Africa, and caused deaths in one of five adults in Europe and North America between the 17 and 19th centuries (Comas et al., 2013). *H. pylori* is a primary cause peptic ulcers and gastric cancer (IARC, 1994).

## Summary

*H. pylori* infection most likely acts as a weak surrogate for the presence of poor hygiene. The hypothesis that *H. pylori* or specific *H. pylori* antigens provide protective antigens reducing the frequence of atopy, allergy, or asthma is not supported by the current data and was falsified in experiments testing projections of the hypothesis. We conclude that *H. pylori* is a major human pathogen that causes progressive damage to the stomach. The infection is etiologically associated with chronic gastritis, peptic ulcer, and gastric malignancies, and should be eradicated.

## Author contributions

MM, IN, DG, and YY contributed to data collection, analysis, and interpretation, and wrote the manuscript. YY and DG revised the manuscript to include important content. All authors read and approved the final version of the manuscript.

### Conflict of interest statement

DG is a paid consultant and has received research funding from RedHill Biopharma regarding novel *H. pylori* therapies and is a consultant to BioGaia regarding use of probiotics for *H. pylori* infections. The other authors declare that the research was conducted in the absence of any commercial or financial relationships that could be construed as a potential conflict of interest.
